# Classical experiments in whole-body metabolism: open-circuit respirometry—diluted flow chamber, hood, or facemask systems

**DOI:** 10.1007/s00421-017-3735-5

**Published:** 2017-10-27

**Authors:** P. F. M. Schoffelen, G. Plasqui

**Affiliations:** Department of Human Biology and Movement Sciences, NUTRIM School of Nutrition and Translational Research in Metabolism, Maastricht University Medical Centre +, PO Box 616, 6200 MD Maastricht, The Netherlands

**Keywords:** Respiration chamber, Energy expenditure, Indirect calorimetry, Whole-room calorimeter

## Abstract

For over two centuries, scientists have measured gas exchange in animals and humans and linked this to energy expenditure of the body. The aim of this review is to provide a comprehensive overview of open-circuit diluted flow indirect calorimetry and to help researchers to make the optimal choice for a certain system and its application. A historical perspective shows that ‘open circuit diluted flow’ is a technique first used in the 19th century and applicable today for room calorimeters, ventilated hood systems, and facemasks. Room calorimeters are a classic example of an open-circuit diluted flow system. The broadly applied ventilated hood calorimeters follow the same principle and can be classified as a derivative of these room calorimeters. The basic principle is that the subject breathes freely in a passing airflow that is fully captured and analyzed. Oxygen and CO_2_ concentrations are measured in inlet ambient air and captured outlet air. The airflow, which is adapted depending on the application (e.g., rest versus exercise), is measured. For a room indirect calorimeter, the dilution in the large room volume is also taken into account, and this is the most complex application of this type of calorimeter. Validity of the systems can be tested by alcohol burns, gas infusions and by performing repeated measurements on subjects. Using the latter, the smallest CV (%) was found for repeated *V*O_2max_ tests (1.2%) with an SD of approximately 1 kJ min^−1^. The smallest SD was found for sleeping metabolic rate (0.11 kJ min^−1^) with a CV (%) of 2.4%.

## General introduction

Indirect calorimetry is the indirect determination of total energy expenditure (EE) of the human body by measuring gas exchange, i.e., O_2_ consumption and CO_2_ production, to derive estimates of quantities of substrates oxidized. The combination of known quantity and energy density of substrates then reveal the total amount of EE. In that regard, ‘indirect calorimetry’ is essentially different from ‘direct calorimetry’, which measures the heat dissipated by the body directly. Several aspects of food composition, oxidation, energy released as heat and work and gas exchange had to be discovered and combined before indirect calorimetry became a practical solution for the measurement of human EE.

Even though most current systems are based on indirect calorimetry, one could state that direct calorimetry is in fact the gold standard for measuring EE as no “indirect” conversion factors are needed to calculate EE values from chemical energy stored in substrates. It is important to understand that energy has many forms, and it can be stored, transferred, and converted in at least as many ways. A few examples are energy in the form of heat, radiation (light), and movement. Energy can be transferred and converted from one form into another, which, for instance, will involve chemical reactions or mechanical work. Hence, a second essential difference between direct and indirect calorimetry is that while direct calorimetry measures heat dissipated by the body, indirect calorimetry measures total EE, and this total energy is not always entirely released as heat from the body. When performing exercise, part of the total EE of the body is used to perform this ‘external work’ and is not released as heat. A good example to illustrate this difference is the paper by Webb on “The work of walking: a calorimetric study” (Webb et al. 1988). During walking on a treadmill, subjects’ EE was measured directly using a suit calorimeter and indirectly using gas exchange. It was shown that during walking, part of the energy used by the body was not released as heat, but that ‘work’ was being done. Please note that work converted to heat inside of a direct calorimeter will be included as measured energy, and energy from external sources (lights, fans, airco, tv, computer) must be excluded. The same holds true for indirect calorimetry regarding changes in gas composition not originating from the body (carbonated drinks, burning cigarettes, and flushing toilet).

This review will focus on indirect calorimetry and, more specifically open-circuit, diluted flow systems.

## Historical perspective

As early as 360 b.c., Plato described ‘breathing’ in his work “Timaeus”, later discussed by his student Aristotle in “On Youth and Old Age, On Life and Death, On Breathing”, herein combining the terms ‘internal fire’ and ‘breathing’ (Aristotle and Ogle 1897). The mysterious relation of food, energy, and breathing caused Aristotle to write “Moreover, in what sense are we to understand this fantastical notion, that heat is generated out of the breath? For it is out of food rather than out of the air that we see heat developed” (Aristotle and Ogle 1897). This sentence comes as close to describing the basis for both direct and indirect calorimetry as was possible at the time.

In 1614, Santorio Sanctorius found a difference in the weight of all foods consumed and excrements produced (Santorio 1614). He referred to this, at that time unexplained difference, as “insensible perspiration”. In essence, Sanctorius had discovered the weight of carbon leaving the body as CO_2_, but could not explain it at the time (Santorio 1614).

**Fig. 1:**
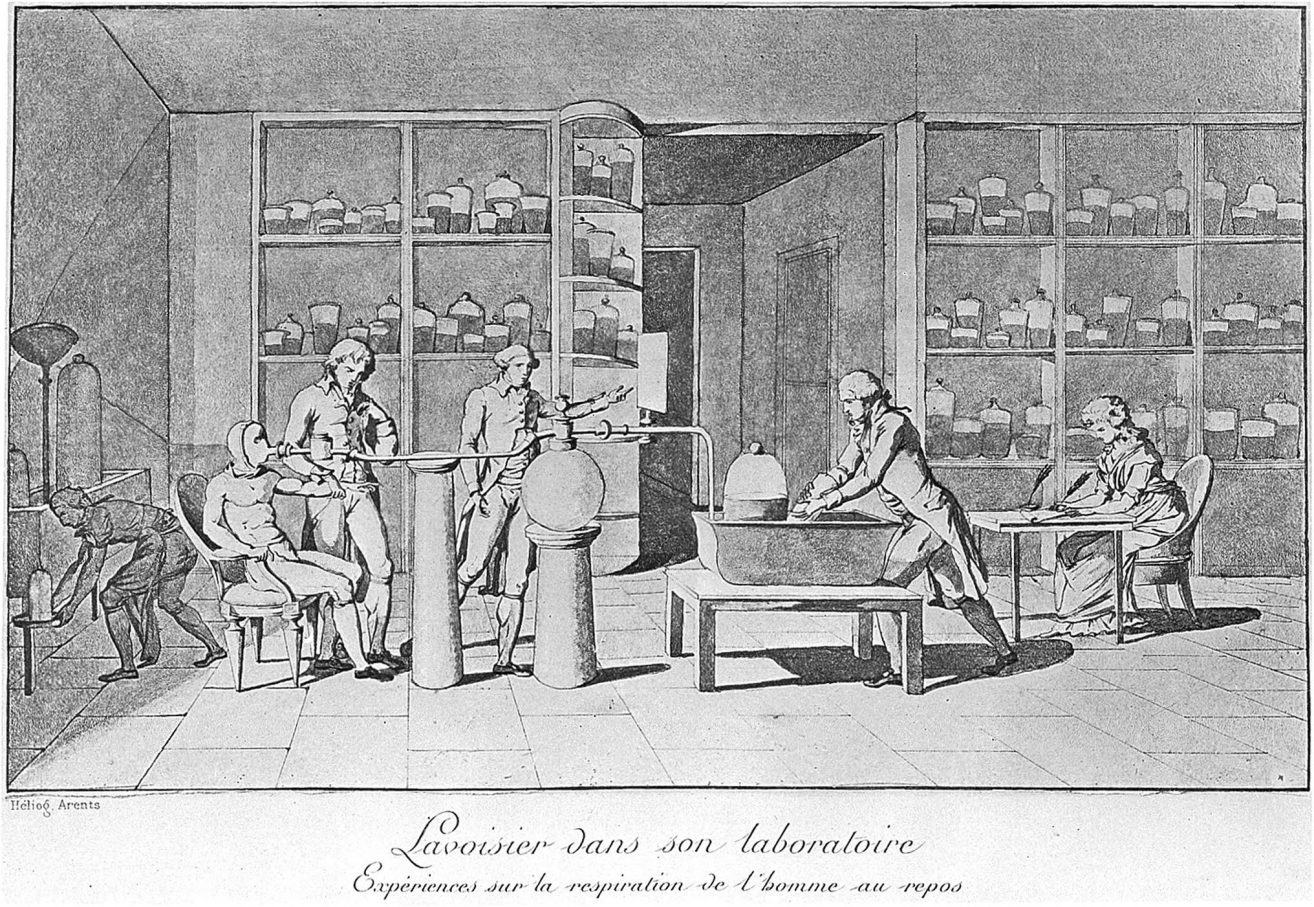
Lavoisier measuring the respiration of a subject at rest, as drawn by his wife. Adopted from: Wellcome Library London (Grimaux 1888)

A next step occurred in 1774 when Priestly found that oxygen in the air was not an unchangeable constant (Priestley 1774), and in the years 1774–1777, discoveries in the field of the respiration of humans and animals by Antoine-Laurent Lavoisier and his wife and coworker followed (Lavoisier 1777). They were able to measure oxygen consumption and carbon dioxide production, yet without knowledge of substrate oxidation and its conversion to EE (Fig. 1) (Grimaux 1888).

Just a few years later, in 1780, Lavoisier and Laplace (Lavoisier 1783) published details on the use of the famous “ice-calorimeter”, enclosing an animal in a sealed and insulated space cooled with ice to reveal its heat production (direct calorimetry) from the quantity of meltwater produced. Thus, Lavoisier probably built both a first direct calorimeter as well as a first indirect calorimeter, even though gas-exchange results of the latter could not yet be converted to EE. Lavoisier concluded “La respiration n’est qu’une combustion lente de carbone et d’hydrogène, qui est semblable en tout á celle qui s’opère dans une lampe ou dans une bougie allumée, et que, sous ce point de vue, les animaux qui respirent sont de véritables corps combustibles qui brûlent et se consument.” or “Breathing is but a slow combustion of carbon and hydrogen, which is similar in all respects to that which takes place in a lamp or in a lighted candle and that from this point of view, animals that breathe are real combustible bodies that burn and consume”, establishing that heat produced by a living being is comparable to the chemical reaction of burning food in fire, though at a slower pace **(**Fig. 1 **)** (Lavoisier 1783).

**Fig. 2:**
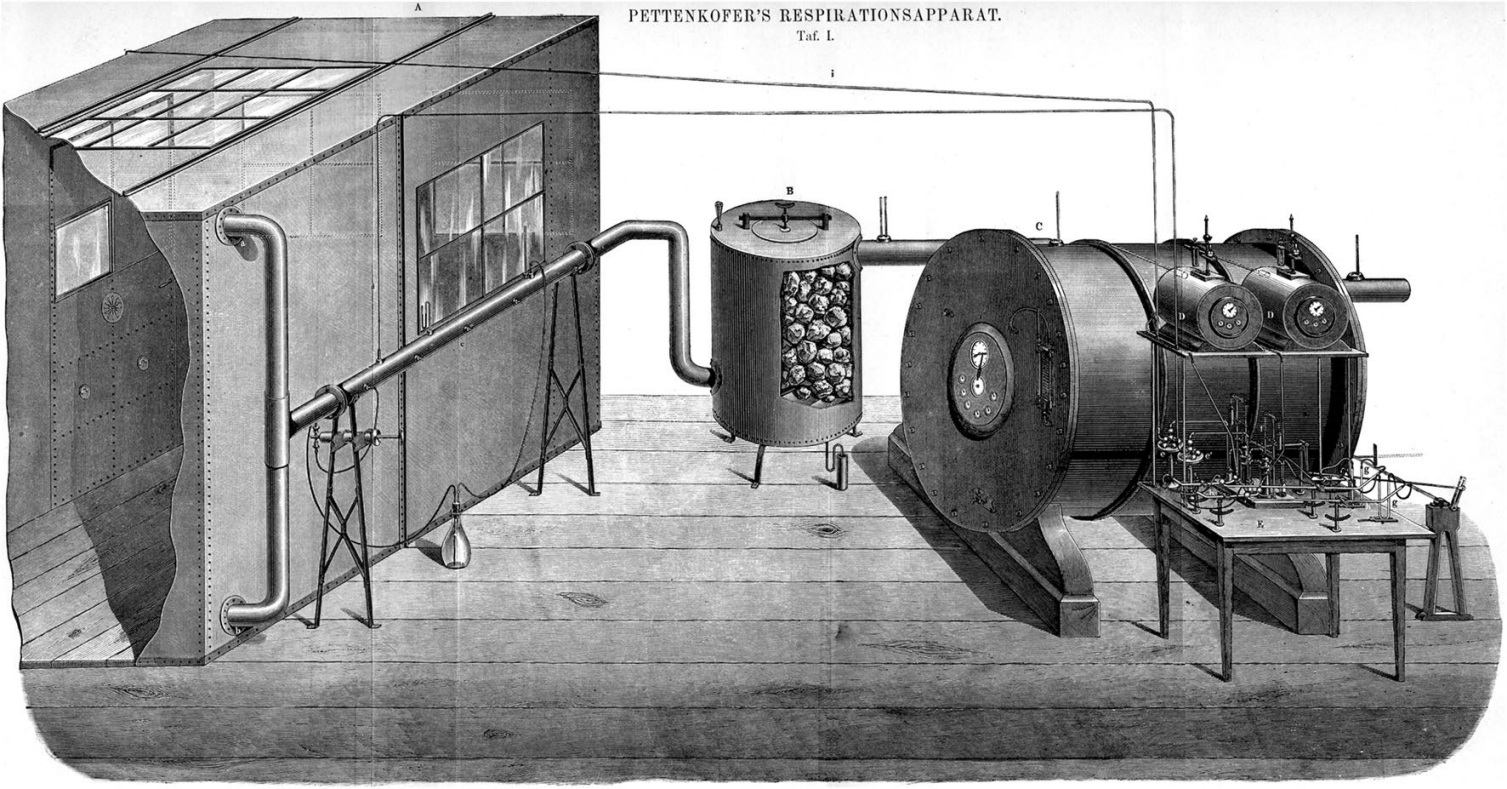
Pettenkofer’s Repirationsapparat, the first human whole-room open-circuit indirect calorimeter. Adopted from: *“Lehrbuch der Physiologischen Chemie”* by Eugen von Gorup-Besanez (Gorup-Besanez 1867)

In the late 19th century, nearly, a 100 years after Lavoisier, the scientific know-how of physics, chemistry, and food composition had progressed far enough to build the first whole-room open-circuit indirect calorimeter, as described by Pettenkofer in 1862 (Fig. 2) (Pettenkofer 1862).

Towards the turn of the century, details on substrate oxidation as published by Magnus-Levy (1893) and Zuntz and Schumburg (1901b); Zuntz and Lehmann (1890) became available. Haldane in 1862 described his apparatus and methods for measuring gas-exchange postulating “it being assumed that no weighable quantities of nitrogen, or any other gaseous or volatile substances, are given off from, or absorbed by, the animal during the experiment” (Haldane 1892). This important postulate regarding conservation of inert gasses in-, or passing through, a calorimeter is known as the “Haldane transformation” or “Haldane correction”. The inert gasses in fresh air, mostly Nitrogen (78%) and small amounts of noble gasses like Argon and Helium (0.9%), are not used in the body. Usually, oxygen consumption is not equal to carbon dioxide production (unless RER = 1), i.e., breathing volume in is not equal to breathing volume out. Haldane states that for inert gasses, volume in remains equal to volume out, and this can be used for calculating changes in breathing volume out from in or vice versa.

**Fig. 3:**
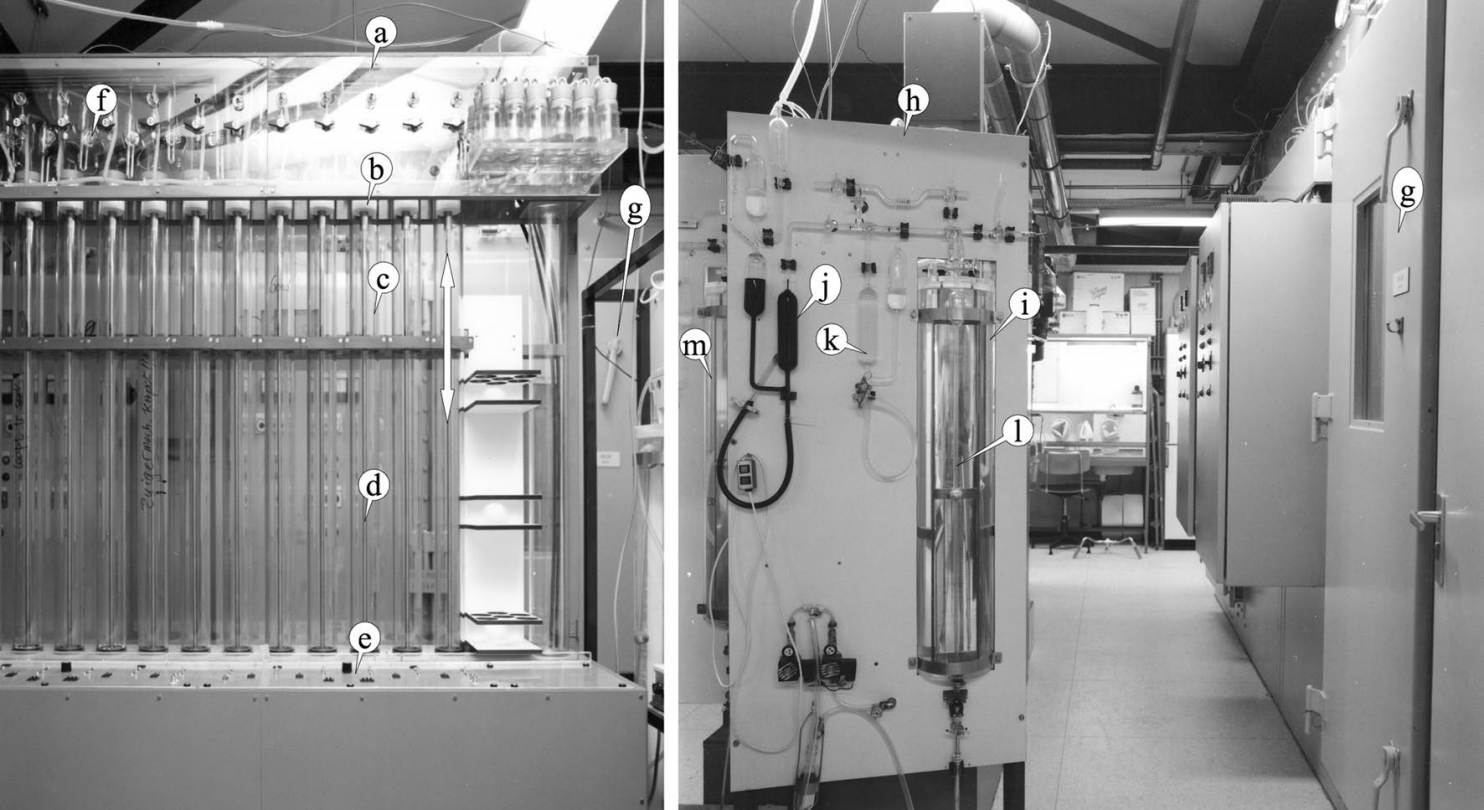
Respiration chamber and chemical gas analysis apparatus as applied at the university of Maastricht in the 1980s. Left panel, *a*) an automated sample unit (*a*), with Teflon and mercury-sealed pistons (*b*) moving up and down (arrow) in precision glass tubes (*c*) by means of a controlled spindle (*d*). The control of timing (*e*) and solenoid valves (*f*) allowed for momentary sampling, metal–glass sealing, and unimpaired storage of gas samples taken from the respiration chambers (*g*). Right panel, a modified Haldane chemical gas analysis apparatus (*h*). Its precision dual cuvettes in water container (*i*) allowed analysis to 0.001% absolute for CO_2_ and O_2_ when applying a three-sample evaluation. It works by removing O_2_ and CO_2_ from the sample cuvette by means of flushing with O_2_ absorbent (*j*) and CO_2_ absorbent (k) and measuring the resulting change in gas volume in the sample cuvette (l). The second unit (m) can be seen behind the front unit, and two units were needed for continuous measurements over more than 24 h

Thus, the Haldane correction allows to achieve near-perfect balance regarding in-flow and out-flow, which is still used in indirect calorimeters to this day. Haldane (Haldane 1898) in 1898 also meticulously described a method for chemical analysis of gas composition, and his method as well as Scholander’s from 1947 (Scholander 1947) and modifications were still in use in the 1980s **(**Fig. 3 **)** (McLean and Watts 1976; Webb et al. 1988; Schoffelen 1985).

In 1904, Atwater augmented a whole-room direct calorimeter with closed-circuit gas-exchange measurements (Atwater and Benedict 1905). This combination of direct- and closed-circuit indirect calorimetry “was a landmark in human calorimetry” as Mclean and Tobin remarked (McLean and Tobin 1987). Many more pioneers of the era exist.

Well into the 19th century, scientists were able to start larger scale research into the relation of work, heat, and respiration in both animals and humans (Pettenkofer 1862; Ott and Foster 1891; Haldane 1892; Atwater et al. 1897; Marcet et al. 1898; Zuntz and Schumburg 1901b; Atwater and Benedict 1905; Hagemann 1911; Langworthy and Milner 1911; Hill and Hill 1914). Importantly, the composition of food from three basic substrates fat, protein, and carbohydrates was analyzed and their chemical reaction with oxygen resulting in carbon dioxide and energy was determined (Zuntz and Schumburg 1901b; Magnus-Levy 1893) (Table 1). Note that variation in substrate composition exists (Livesey and Elia 1988).

**Table 1:** O_2_ consumption (l), CO_2_ production (l), and EE (kcal) per gram of substrate used (i.e., carbohydrate, protein, and fat), including correction of EE (kcal) per gram of urinary nitrogen katabolized

Substrate 1 g	O_2_ uptake (l)	CO_2_ production (l)	EE (kcal)	EE (kcal)
Carbohydrate	0.8288	0.8288	4.182	Zuntz 1897 (Zuntz and Schumburg 1901b)
Protein	0.967	0.7752	4.316	Loewy-Lusk 1928 (Lusk 1928)
N_2_-urine katabolized	–	–	− 2.17	Weir (1949)
Fat	2.0193	1.4311	9.461	Cathcart 1931 (Cathcart and Cuthbertson 1931)

This allowed calculation of human EE from gas exchange including the variable contribution of carbon dioxide production and the correction for energy loss of N_2_ compounds in urine (Table 1) (Schoffelen and Plasqui 2016; Livesey and Elia 1988; Consolazio et al. 1963; Brouwer 1957; Weir 1949; Zuntz and Schumburg 1901b). One of the resulting formulae for converting gas-exchange values into human EE was written as

$${\text{EE }}\left[ {{\text{kcal}}} \right]{\text{ }}={\text{ 3}}.{\text{941 }} \cdot {\text{ }}{{\text{O}}_{\text{2}}}{\text{ }}\left[ {\text{l}} \right]{\text{ }}+{\text{ 1}}.{\text{1}}0{\text{6 }} \cdot {\text{ C}}{{\text{O}}_{\text{2}}}{\text{ }}\left[ {\text{l}} \right]{\text{ }} - {\text{2}}.{\text{17 }} \cdot {\text{ }}{{\text{N}}_{\text{2}}}{\text{ }}\left[ {\text{g}} \right].$$

(Weir 1949)

This calculation of EE from gas exchange does not involve measuring heat. It is completely based on measured gas quantities for oxygen consumed and carbon dioxide produced. In the absence of any “direct” measurement of heat, this method based on gas exchange became known as “indirect calorimetry”.

For most of the 20th century, measuring human EE with direct- and indirect calorimeters remained a demanding effort. Computers and electronic equipment were not available or in early development stages. Analysis of gasses required specialized technicians performing chemical procedures to determine values for a single gas sample at a time (Scholander 1947; Haldane 1898), while sensors were manually logged or plotted using chart recorders.

These efforts of reputable research groups resulted in truly excellent work, for the greater part still applicable today, having provided validated methods for heat- (direct) and gas-exchange (indirect) measurements, measured caloric values of the macro-nutrients protein, carbohydrates and fat, as well as formulae for EE calculations from gas-exchange and measured urinary nitrogen (Harris et al. 1919; Lusk 1917; Zuntz and Schumburg 1901b; Haldane 1892; Pettenkofer 1862; Webb 1985).

In the 1960s, developments in electronics started a revolution in equipment, and this also resulted in the first mini- and micro-computers of the 1970s. Research groups focussing on Human EE in Cambridge (Dauncey and Murgatroyd 1978) and Lausanne (Jequier et al. 1978) were among the first to take advantage of these developments, followed within a decade by many others (Seale et al. 1991; Hill and Sun 1991; Rumpler et al. 1990; Charbonnier et al. 1990; Frappell et al. 1989; Webb et al. 1980, 1988; Shetty et al. 1987; de Boer et al. 1986, 1987; Ravussin et al. 1986; Garrow and Webster 1986; Schoffelen 1985; Garby and Lammert 1984; Jequier and Schutz 1983; Aulick et al. 1983; Schutz et al. 1982). This increase in indirect calorimetry accelerated with the advent of commercial metabolic carts, bringing ventilated hood diluted flow indirect calorimetry to the bed side. This proliferation as well as technical developments did not change the basic calorimetric principles applied, yet the field of human indirect calorimetry was vastly expanded.

In recent literature, no operational larger whole-room calorimeters with both direct- and indirect calorimetry could be found; they seem all to have been decommissioned, and the last operational whole-body direct calorimeter (Kenny et al. 2017) may be the re-engineered Snellen air-flow calorimeter in Ottawa, Canada (Reardon et al. 2006). In contrast, whole-room calorimeters with indirect calorimetry have in part remained operational and in part were newly constructed. An estimate of operational whole-room indirect calorimeters is about 12–15 from literature and 10–15 more known to be in existence, effectively steady 20–30 sites worldwide.

A trend in whole-room indirect calorimetry that as yet has to realize its full potential and become a common application is the search for fast dynamic response, providing a high degree of short interval and full-range accuracy. Whole-room indirect calorimeters have a reputation for slow response, as accuracy is dependent on the multiplication of accuracy of gas analysis with a large volume and thus problematic for very short interval evaluation. Technical advances and computers have resulted in a typical 15–30-min interval evaluation (Webb et al. 1988; Nguyen et al. 2003; Henning et al. 1996; Tokuyama et al. 2009), where accuracy for low-level EE remains dependent on the minimum floor-level noise of the individual implementation (Murgatroyd et al. 1987). The individual implementation may cause unfiltered noise levels in gas-exchange values varying from 50 to 1000 ml min^−1^ peak to peak (personal observation and communication), and the search for fast dynamic response has mostly been focussed on mathematical procedures for re-evaluation of measured results, i.e., noise reduction (Tokuyama et al. 2009; Granato et al. 2004).

It is surmized that a next step in indirect calorimetry will combine improved measured accuracy for gas analysis and volumetric aspects, with mathematical handling of the smallest finite-step evaluation. To achieve this next step, the classic focus on physics must be combined with modern day computer technology, i.e., attention to input (measurements) must match the current attention to mathematics.

## Types of open circuit indirect calorimeters

**Fig. 4:**
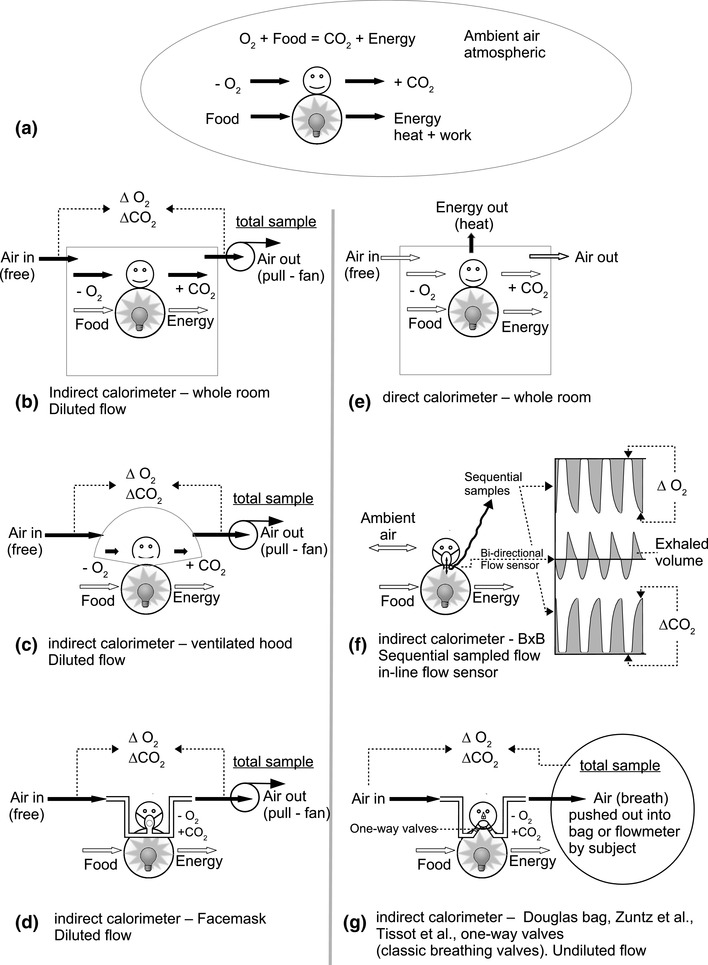
Graphical overview of calorimetry showing: **a** production of energy and CO_2_ by human slow combustion of consumed food with consumed oxygen. The next two graphs show whole-room indirect calorimetry (**b**) and direct calorimetry (**e**) with respective measured parameters highlighted as black arrows. The indirect calorimeter (**b**) is drawn as a pull system with the air drawn through the room. This is near-identical to the common setup for a smaller volume ventilated hood system (**c**), and can also be applied with a facemask (**d**) connected to an airflow without flow sensor (**f**) or breathing valves (**h**). The depicted calorimeter types b, c, d, and e allow free breathing with enforced ventilation (pull fan) and these dilute the breathing in a larger volume or volume over time (flow). The two others (**f, g**), respectively, depict the use of an in-line bi-directional flow sensor with a single sample line transporting sequential samples of inhalation and exhalation (**f** breath by breath or B×B) and **g** use of classic one-way breathing valves to aggregate total exhalation in a Douglas bag (Douglas 1911) or totaling flowmeter (Zuntz and Schumburg 1901b; Tissot 1904). These last two work with the total air flow powered by the breathing of the subject

In principle, there are different basic types of indirect calorimeters to choose from recognizable in the way that subjects are connected and how breath is collected and analyzed. Commonly during exercise, and sometimes during rest, a fixed connection to the face is used (Fig. 4d, f, g). This can be either a facemask (Fig. 4d, f) or a mouthpiece with nose clip (Fig. 4g). In this paper, the term facemask implies the possible application of a mouthpiece unless noted otherwise. With a facemask, three different ways of capturing exhaled air can be defined: (1) full capture of non-diluted exhaled breath (Fig. 4g) or (2) full capture of exhaled air, diluted in a bypass flow (Fig. 4d) or (3) breath-by-breath (BxB) analysis for a partial sample of exhaled air (Fig. 4f). For measurements of basal (or resting) metabolic rate, usually, a ventilated hood system is used (Fig. 4c). A small confined space in the form of a clear-plastic hood is placed over the subject’s head, allowing full capture of exhaled air diluted in a bypass flow drawn through the hood. For measurements of EE over longer intervals, a whole-room calorimeter also referred to as respiration chamber is used (Fig. 4b). In this case, exhaled air is fully captured and diluted in both a bypass flow and a large volume (room). Hence, the use of full capture of exhaled air in a diluted flow is applicable to facemasks, ventilated hoods, and respiration chambers (Fig. 4b–d).

With the theoretical exception of breath-by-breath systems, most indirect calorimeters will measure over a longer “aggregation” interval, i.e., over multiple breaths. This is typical for full capture of exhaled air, rendering assumptions for synchronization of breathing and gas analysis unnecessary. This may be considered an advantage due to the fact that synchronization is not easily proven or validated, specifically for the higher breathing frequencies (Proctor and Beck 1996; Yamamoto et al. 1987; Noguchi et al. 1982; Wessel et al. 1979; Perret and Mueller 2006; Larsson et al. 2004; Prieur et al. 2003). A disadvantage is that measurement of a number of breaths over an aggregation interval in a small volume may have an uncertainty of ± 0.5 breaths, unless breathing is measured for synchronization. Hence, slow breathing frequencies at rest may result in visible peaks and valleys of EE.

Since tidal volume of breathing is not constant (Aliverti et al. 2004; Potter et al. 1999; Wessel et al. 1979) even breath-by-breath measurements will show variation and require multiple breaths over an aggregation interval. For larger volume (room) calorimeters, the impact of breathing is supposedly insignificant as the individual breaths average out in the larger volume.

## Open-circuit diluted flow calorimeters

### Principle

Indirect calorimeters using open-circuit diluted flow respirometry are in essence not different from other open-circuit respirometry applications except for measuring diluted breathing in a larger volume in contrast to measuring undiluted subject exhalation by means of a mixing chamber, Douglas bag or by measuring undiluted breath samples at the mouth (breath-by-breath systems).

The best-known open-circuit diluted flow calorimeters are most whole-room calorimeters or respiration chambers, while the most widely found are the many ventilated hood applications. In all instances, a continuous flow of fresh air is directed through the chamber or hood or facemask and all in- and outgoing air is analyzed for O_2−_ and CO_2−_ concentrations, while the flow through the system is measured (Fig. 4a–c).

A respiration chamber measures EE over longer stay intervals, comfortable and without hindrance during sleep or activities. In contrast, the less comfortable facemask provides measurement of EE for intervals as small as a practical 30 s. A ventilated hood may be considered an intermediary type between a whole room and a facemask, allowing bedside application while providing the minimum comfort required for measurement of BMR.

An essential parameter distinguishing these calorimeters is the volume in which the exhaled air is captured. The volume of a ventilated hood system is about 25–40 l with a typical air flow-through of 60–100 l min^−1^ for normal weight adults, whereas a whole room has a volume of 5000–30,000 l with a typical air flow-through in the order of 100 l min^−1^. Using a facemask, the volume of the mask is very small and the flow of air past the mask may vary from < 100 l min^−1^ for resting or low activity EE up to > 500 l min^−1^ for maximal exertion testing (*V*O_2_max). Volume or volume over time, i.e., flow, is an important parameter for calculating EE using indirect calorimeters. At this instance, it should be noted that any volumetric expression is dependent on temperature, pressure, and often humidity content. Well-known denominators are ATP, BTP, and STP for ambient, body, and standard temperature and pressure, respectively, and these are complemented with saturated (S) or dry (D) regarding water vapour content (no S or D indicates an unspecified percentage of water vapour, possibly measured). Thus, STPD is a dry volume at standard 0 °C and 1013.25 mBar, while BTPS is a saturated “wet” volume at 37 °C body temperature and the prevalent ambient pressure (measured). These volumetric measures are very different and must be correctly defined and measured. Typically, diluted flow calorimeters will correct all volumetric measures to STPD notation, while non-diluted flow calorimeters may use an ATPS assumption for Ve, i.e., expired “wet” air at 37 °C.

Another volumetric aspect is dead space. An advantage of open-circuit diluted flow indirect calorimetry is that a grander stream of the air passes the subject without in-line flowmeter or breathing valve(s), providing the shortest possible open pathway between mouth and fresh air volume or flow. An artificial pathway and/or resistance in-line with subject breathing flow can affect the performance of subjects. The volume of the small pathway or “dead space” artificially lengthens the anatomical dead space, i.e., the part of the airway that does not participate in actual breathing, and effectively decreases alveolar ventilation (the active ventilation of the lung).

In addition, any artificial resistance to flow will be added to the anatomical airway resistance, increasing the required work for breathing. For EE measurement with indirect calorimetry, the best practice is to decrease, or circumvent, breathing dead space, and resistance. In this respect, elimination of flowmeter or breathing valves in-line with subject breathing will decrease, or near enough eliminate, aspects of dead space and resistance.

In Fig. 4, three types of calorimeters that fit the description of open-circuit diluted flow indirect calorimetry are depicted (Fig. 4a–c). For reference, a direct whole-room calorimeter (Fig. 4e), a breath-by-breath indirect calorimeter (Fig. 4f), and a classic non-diluted calorimeter using breathing valves with Douglas bag (Douglas 1911) or flowmeter (Zuntz and Schumburg 1901a; Tissot 1904) (Fig. 4g) are depicted as well. A closed-circuit calorimeter is not drawn.

Placed in historical context, the first indirect room calorimeters captured all exhaled breath and analyzed its chemical composition, this is best represented in Fig. 4b. For non-diluted flow capturing all exhalation (Fig. 4g), this is best comparable to the setup first used by Lavoisier (Fig. 1).

### Respiration chambers or whole-room calorimeters

**Fig. 5:**
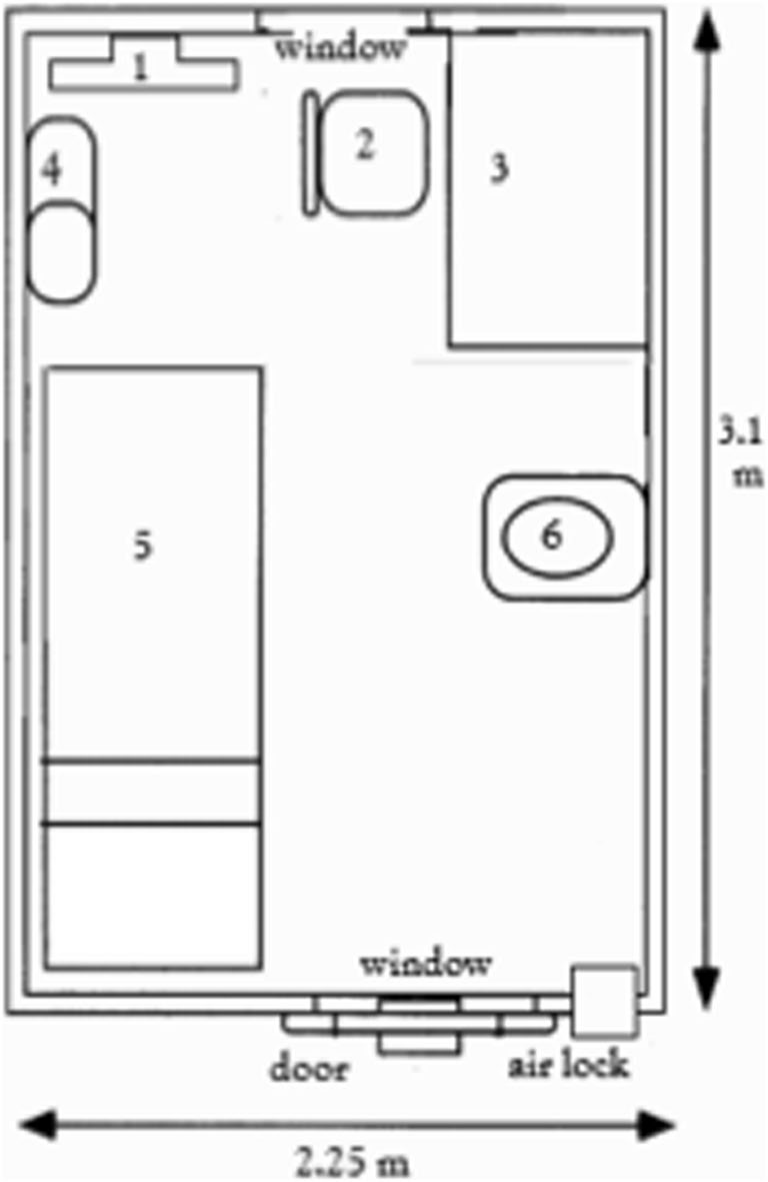
Schematic representation of a typical room-calorimeter layout as currently being employed at the Metabolic Research Unit Maastricht, The Netherlands

A respiration chamber is used for measuring human EE over longer periods of time, typically between 12 h (e.g., overnight for measuring sleeping metabolic rate) and 36 h (nigh-day-night) and up to several days. The advantage of a chamber compared to a ventilated hood or facemask is that the subject can move freely within the chamber and that 24-h EE can be measured capturing all the different components of EE, i.e., sleeping metabolic rate (SMR), basal metabolic rate (BMR), diet-induced thermogenesis (DIT), and activity-related energy expenditure (AEE). The latter is of course limited due to the confined spaces of the chamber and is not representative of daily life AEE. To better mimic daily life circumstances in terms of total EE, an activity protocol can be introduced (e.g., by adding a bicycle to the room). A typical respiration chamber includes a bed, table, chair, (freeze) toilet, sink, TV, airlocks and sample ports, intercom, phone, and computer **(**Fig. 5 **)**. For privacy reasons, windows should have curtains and camera’s (if present) an off-switch.

#### Capturing the different components of EE in a respiration chamber

**Fig. 6:**
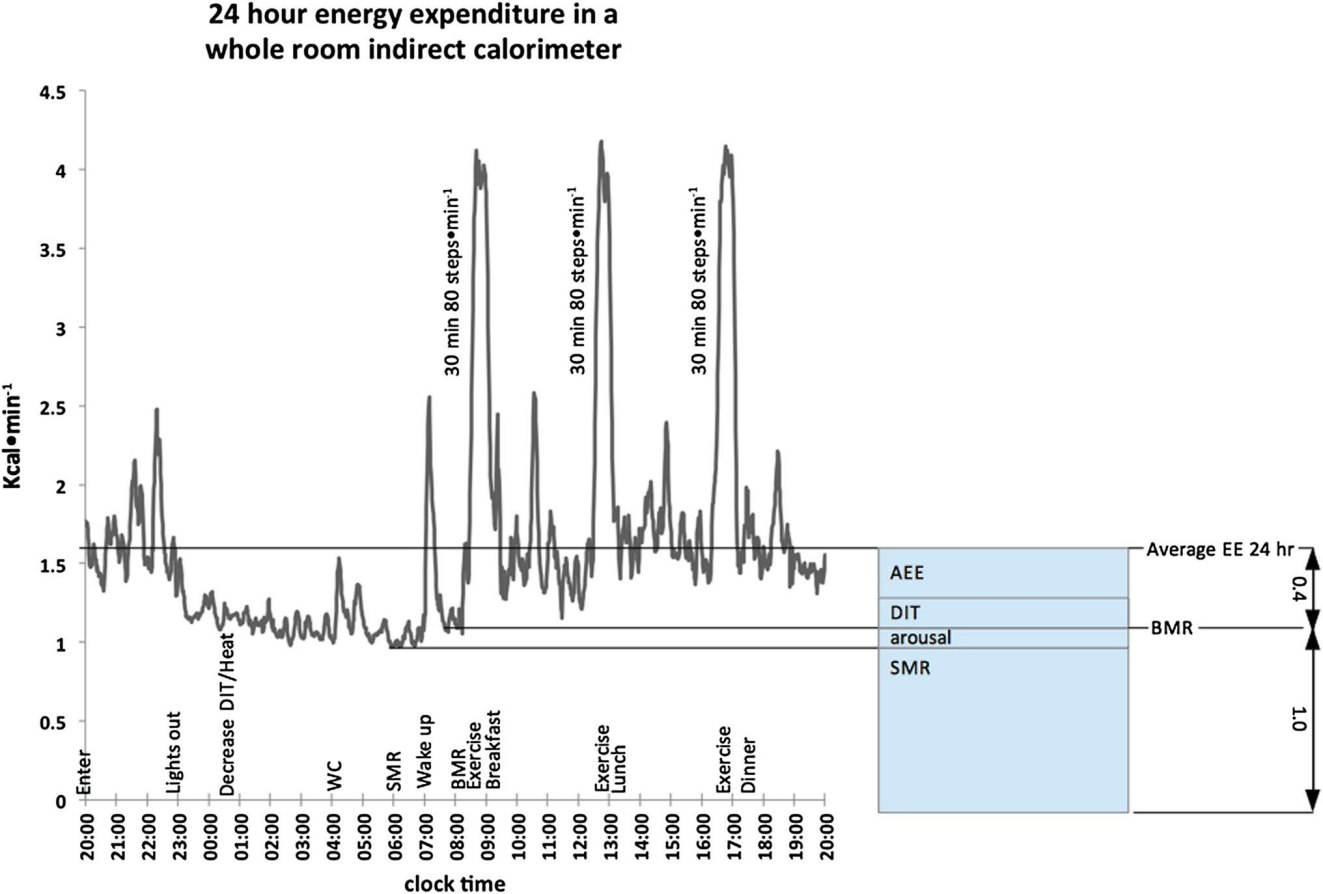
Different EE components as measured during a 24-h stay in a respiration chamber. The protocol is noted on top of the *X*-axis clock time, and subject enters at 20:00 h and stays for 24 h. During the night, EE decreases as DIT and body heat decrease; SMR is determined in the last part of the sleeping period. After wake-up, washing and brushing teeth a rest on the bed (aroused, no sleeping) allowed the measurement of BMR. In daytime, three exercise bouts of 30-min stepping at 80 steps min^−1^ were performed, and each followed by a meal. The average EE over 24 h can be seen to be 40% above BMR and results from diet- and activity-related thermogenesis. A physical activity factor of 1.4 was registered which is typical for a stay in the limited space of a respiration chamber (Westerterp and Kester 2003)

Human EE is not constant, and it varies with time of day, level of activity, food digestion, and other parameters. For each point in time, the momentary EE measured equals the sum of EE components. Although these EE components are never measured separately, some of them can be individually identified over a specific time interval, for instance, during sleep, rest, activity, meals, or over 24 h (Fig. 6).

These individual EE components do not simply start and stop. Each component will have an individual response that can be delayed after the start and/or remain active after the end of the interval for a period ranging from minutes up to hours or even days. Given these time-course effects, the protocol used is of significant importance for the analysis of EE components regarding reproducibility, as well as for allowing comparison of results to those found in the literature. For example, overnight sleep EE values for an 8-h interval (23:00–07:00) will be significantly different from EE values of sleep (SMR) taken as the lowest observed EE over a 3-h interval during the night, typically between 3:00–6:00 in the morning (Schoffelen and Westerterp 2008). The same holds true for BMR and resting metabolic rate (RMR), both providing information on EE at rest, but BMR is more strictly defined then RMR. BMR is measured early in the morning after an overnight fast while lying awake in supine position and under thermoneutral conditions. RMR also indicates an EE level of rest, typically when fasted for several non-active hours. However, position, time of day, and other factors may differ. For example, RMR could be measured several hours after lunch in a sitting position with the subject having been active in the morning, and consequently, RMR will be significantly elevated in comparison with BMR.

A typical pattern of EE over the day as measured in respiration chamber is depicted in Fig. 6, showing the different components SMR, BMR, and average EE over 24 h. DIT and AEE are difficult to separate from each other as both occur simultaneously during the course of the day and cannot be determined without measuring additional parameters. This particular experiment shows the subject going to the toilet in the middle of the night, illustrating how SMR can be disturbed if activity is not taken into account; a typical example of biological variation is even found in a strongly controlled environment.

More sources of biological variation exist, for instance, seasonal or climate influences, health issues, abnormal activity levels, diet, travel, age, gender, body composition, genetics, choices for repeated visits, etc. (Claessens-van Ooijen et al. 2006; Schols et al. 1992; Plasqui and Westerterp 2004; Plasqui et al. 2003; Dugas et al. 2009; van Dale et al. 1989; Van Dale et al. 1987; Donahoo et al. 2004; Blaak and Saris 1996; Webb 1993; Fredrix et al. 1990, 1991a, b, c; Brouns et al. 1989).

Knowledge of biological variation is of importance to the design of studies, typically determining the effect of an intervention, i.e., imposed variation, in one group and comparing it to the results of a group without intervention (van Dale et al. 1989; Van Dale et al. 1987; Brouns et al. 1989; Westerterp-Plantenga et al. 2004, 2009; Smeets and Westerterp-Plantenga 2008; Lejeune et al. 2003).

An important aspect of this variety in EE components is the accuracy and measurement interval in relation with the level of EE. An indirect room calorimeter will have a base level of accuracy that is nearly constant and independent of the level of EE, a floor noise level determined by accuracy of analysis and volume of the calorimeter (Murgatroyd et al. 1987). It is a constant that can be divided over the measurement interval i.e., large for small interval and decreasing over time. A second component is a similar floor noise level originating from the throughput flow and its analysis, constant over time, i.e., increasing as a summed total over time. Both are independent of the actual level of EE, except that in practice, flow may be set at different rates for different levels of EE. Given a medium yet constant flow setting, the floor-level accuracy is near constant, and the absolute SD for results is similar for low-level EE and high-level EE. This means that high levels of EE will have a smaller percentage of error than low levels of EE, or can be measured over a shorter interval with the same accuracy as low levels of EE over a longer interval. This aspect must be taken into account when determining the measurement interval for different EE components. For instance, the lowest EE level of sleep typically requires an interval of several hours (Schoffelen and Westerterp 2008), while higher levels may easily be determined over a 20-min interval (Webb et al. 1988; Nguyen et al. 2003).

#### Push versus pull systems

In Fig. 1b, an indirect whole-room calorimeter was depicted as a pull system, drawing air through the volume. A push system can be drawn near-identical by placing the enforcing fan at the air inside, pushing ambient air into the volume.

Both types exist and have their specific advantages and disadvantages. To explain the pro’s and con’s, let us first envision a system without push or pull flow, simply an enclosure without openings. If the enclosure is 100% airtight, all gas exchanges of a subject inside will integrate over time, showing a decrease in O_2_ and an increase in CO_2_ co-linear with the EE of the subject and effectively this creates a closed-circuit calorimeter for a limited time interval. To remove heat and moisture an air conditioning is needed, just as in calorimeters of the push and pull type. The heating and cooling expands and shrinks the air inside, causing pressure inside to increase and decrease accordingly. If the enclosure is not fully airtight, an increase in pressure will cause air to escape and a decrease of pressure will cause air to be drawn in. Effectively, the air conditioning will cause a bi-directional flow, sequential out-flow and in-flow, trough leakages and the co-linearity of the O_2_ and CO_2_ curves with EE of the subject is compromised.

Most room-calorimeter enclosures are not fully airtight, and the choice between push and pull is also a choice for how leakage is handled. The push type forces air into the enclosure to eliminate other sources of in-flow, while the pull type draws air out of the enclosure to eliminate other sources of out-flow. Both push and pull operations remove the bi-directional nature of leakage. The push and pull flows may be further modulated by the air conditioning unless a pressure equalization bellow is present. Both push and pull systems seem near-identical with a respective positive or negative pressure in the room, and results may be fully identical. Depending on the individual construction, a significant difference can occur due to the composition of the air that is leaked: a push-type calorimeter must assume that all subject air is fully mixed and sampled before air leaves the enclosure through leaks and output. Effectively, the output and leaks need to have the same gas concentrations. A pull-type calorimeter must assume that air surrounding the room (e.g., air in the laboratory, where the room calorimeter is situated in) is identical to inlet air (e.g., air drawn from outside air on top of the building); effectively, the input and leaks need to have the same gas concentrations. Both types assume that either input or output flow is exactly known, while the other side effectively consists of the sum of flow plus a small amount of leakage. This works as indirect calorimeters use a single main flowmeter at input (push) or output (pull) and the Haldane correction (Haldane 1892). The Haldane correction assumes that Nitrogen in the air (inert gas) is not used passing though the calorimeter: flow of N_2_in is equal to flow of N_2_out. For a pull system, measuring the amount of N_2_out also defines the amount of N_2_in, and this N_2_in is the sum of N_2_ trough inlets, i.e., including N_2_in through leakage. For a push system the in’s and out’s are exchanged. The Haldane correction thus allows the use of a single flowmeter (or correct a second one) for either in- or out-flow. This is important as any small difference between individual flow measurements of in and out would otherwise be summed over time. In practice, there would not be a perfect balance. Moreover, the Haldane correction allows for a small amount of leakage in one direction to be accounted for as if it was legitimate flow. In this case, a requirement is that gas composition of leakage is identical to the composition of the Haldane-corrected flow.

Since subjects are allowed to move around inside a whole-room calorimeter, the subject exhalation may or may not be fully mixed if the subject is breathing near a leak, this is a matter of design of the calorimeter, and breath leaked before mixing is hence not being measured to calculate EE. However, a pull system will show the identical effect if the surrounding air is not free of human contamination, visitors or personnel breathing near a leak may have part of their breath drawn in and this part will be added to the EE of the subject. A typical place where this might occur for both push- and pull system is the blood-sampling port, where during sampling the subject is facing a possible leak as well as the person taking the sample at the outside.

Regarding the impact of leakage, a pull system may pose less demands on mixing and sampling in comparison with a push system, as the pull system draws all subject air through one outlet. In contrast, a push system will allow flush toilet and other amenities that lose a small amount of the air, all of which must be accounted for or eliminated in a pull system.

Importantly, the above-described effect of personnel surrounding a pull calorimeter may also disturb ventilated hood measurements in the close presence of personnel. These small pull systems draw in and sample surrounding air. If ambient air is infrequently sampled and/or the ambient air sample line is placed too far from hood air inlet, the variation of ambient gas composition will affect the EE result.

#### Sources of error

In human indirect calorimetry, a vast amount of sources of errors can be identified (McLean and Tobin 1987). A non-exhaustive sample of common sources of errors can be subdivided in theoretical, system (random and systematical), and practical errors.

Theoretical errors originate for the greater part from choices in assumptions and formulae. A few typical assumptions are the absence of leakage, all volumes of gasses are fully mixed before evaluation, all gasses are ideal gasses, and inert gasses pass through the calorimeter unchanged (Haldane correction). A typical choice of a formula is the one used for conversion of gas-exchange results to EE (Weir 1949; Brouwer 1957) that is based on the composition of substrates (Livesey and Elia 1988), which is assumed to be identical to those consumed by the subjects at hand.

In this context, a further requirement is that validation tests are suitable for mimicking the biological application. For instance, it should be realized that when checking a breath-by-breath (BxB) system with alcohol combustion in diluted flow mode, the BxB measurement is a different mode compared to the diluted flow (ventilated hood) mode.

System errors originate from the technical implementation of the calorimeter, and these errors can be random or systematical. The former average out over time, while the latter may create a larger summed error over time.

Some examples are measurement noise and drift, aspects of digitalization, flushing of sample system and clearing of residue, accuracy of calibration, and incorrect validation methods (i.e., not suitable for the biological application). A biological systematical example is the effect of skin diffusion of CO_2_ (Livesey and Elia 1988; Alkalay et al. 1971; Fitzgerald 1957; Klocke et al. 1963), which accounts for a + 1–2% difference in measured CO_2_ for whole-room calorimetry versus ventilated hood or facemask calorimetry.

Practical errors origin from the application of the calorimeter, and these can be operator errors as well as deficiencies in locality or supplies. Examples are failure to adhere to protocol, for example, by incorrectly closing the calorimeter or other sources of leakage, or by incorrect setting of flow through the calorimeter causing a too large or too small dilution flow. Localities may impact temperature or ambient air composition, and required supplies such as calibration gasses that may have an incorrect value or certificate accuracy.

A typical source of error has been highlighted above for Push versus pull systems, leakage, and contamination of inlet air. An indirect calorimeter measures air quantities and differences in air composition caused by a single subject. No subject air may go unaccounted for and no air from others may be incorporated at all.

It can be speculated that proliferation of small indirect calorimeters (metabolic carts) and a co-occurring decrease in required effort to perform experiments, also decreased availability of expert know-how at the numerous sites applying indirect calorimetry. In some instances, an indirect calorimeter may be perceived as a magical black box for measuring human EE. However, it remains a sensitive measurement. For example, an acceptable accuracy for BMR measurements may be ± 5%, corresponding to ± 10 ml min^−1^ O_2_-consumption, typically measured with a ventilated hood with a flow of ≥ 40 l min^−1^. However, this minimum demand of only ± 5% biological accuracy requires ≤ 0.025% (i.e., ≤ 10 × 40,000^−1^) overall technical accuracy. This large difference between biological and required technical accuracy can be confusing and causes an underestimation of required technical effort.

For sites applying whole-room calorimetry, with its further increased sensitivity to errors, the required technical effort will become elevated to a degree, where staff have gained sufficient know-how and experience before achieving operational status at even a basic level.

Note that experience can also have its negative side-effects, as demands on technology change, so must procedures and understanding. It is possible to have proven operating procedures, and simply fail to observe a drift in an apparatus as it constantly remains within the old “approved” error range. A practical example would be a calorimeter supposed to operate within 5% absolute error defined as 1 ± 2% (mean ± SD). Suppose that SD improved from 2 to 0.5%; now, a 3 ± 0.5% (mean ± SD) may pass inspection, while an update in procedures should in fact keep it within 1 ± 0.5%, i.e., within 2% instead of 5% maximum error range.

## Methods for system validation

### Calibration is not validation

When using a calorimetry system, it is utterly important to perform regular validity testing. Validity testing is not to be confused with calibration. First, the different components inside the calorimeter (e.g., flowmeter, pressure sensor, analyser) may have been calibrated and found suitable for the intended use, but that does not test the functioning of the entire system as a whole. Second, the analysers should be calibrated each day (or even more frequent) before being used to measure a subject. This can be done by consecutively flowing two gasses containing a known concentration of O_2_ and CO_2_ through the analysers (dual span), one of which may well be 100% N_2_ gas (zero for O_2_ and CO_2_), or use a single gas and ambient air. The latter assumes a typical 20.9% oxygen in ambient air and commonly filters out the 0.04% ambient CO2 for creating a zero gas for CO2. Besides this analyser calibration, several options are available and required to test validity of the system, which are described below.

### Alcohol combustion

**Fig. 7:**
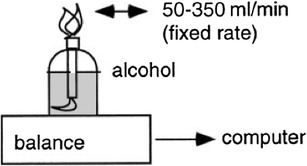
Indirect calorimeters may be validated by combustion of alcohol. The amount of combusted alcohol is calculated from weight change of the burner. Connecting the balance to a computer allows continuous measurement of rate of combustion

A typical validation method for an indirect calorimeter is the combustion of a measured quantity of a high-purity fuel, such as methanol or ethanol. The combustion of 1 g of such a fuel will require a known amount of O_2_ and produce a known amount of CO_2_. Measured gas-exchange values from the calorimeter can then be compared to the known values based on the chemical process for the amount of fuel combusted. The most common method uses an alcohol burner, burning high-purity ethanol, or methanol (Fig. 7) inside the calorimeter. For hood systems, the standard hood is replaced by a different one (more heat resistant) to allow for the burning flame and its heat. This method also challenges the capability of the calorimeter to cope with both H_2_O and heat produced.

### Gas infusion

**Fig. 8:**
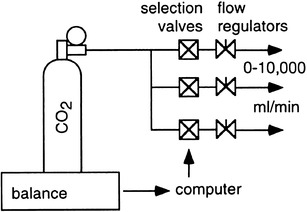
Indirect calorimeters may be validated by gas infusion. The amount of injected gas is calculated from weight change or from volumetric flow. A computer may allow for continuous registration and change in rate of infusion

A second method is the infusion of a measured quantity of a high-purity gas, and gas-exchange results of the apparatus must match the amount infused. This process does not produce H_2_O or heat, though cooling due to adiabatic expansion may occur (Fig. 8).

The match between infused gas and gas-exchange measurements is easily understood for CO_2_. An amount of CO_2_ infused into the calorimeter must register exactly as if it originated from a subject, and at the same time, the O_2_ uptake must remain zero as the total of CO_2_ infused should be accounted for.

The simulation of O_2_ consumption is more complex as there cannot be a “negative” infusion of O_2_ to simulate O_2_ uptake. Instead, an inert gas, typically N_2_, is infused, diluting the O_2_ concentration in the outgoing airstream.

**Fig. 9:**
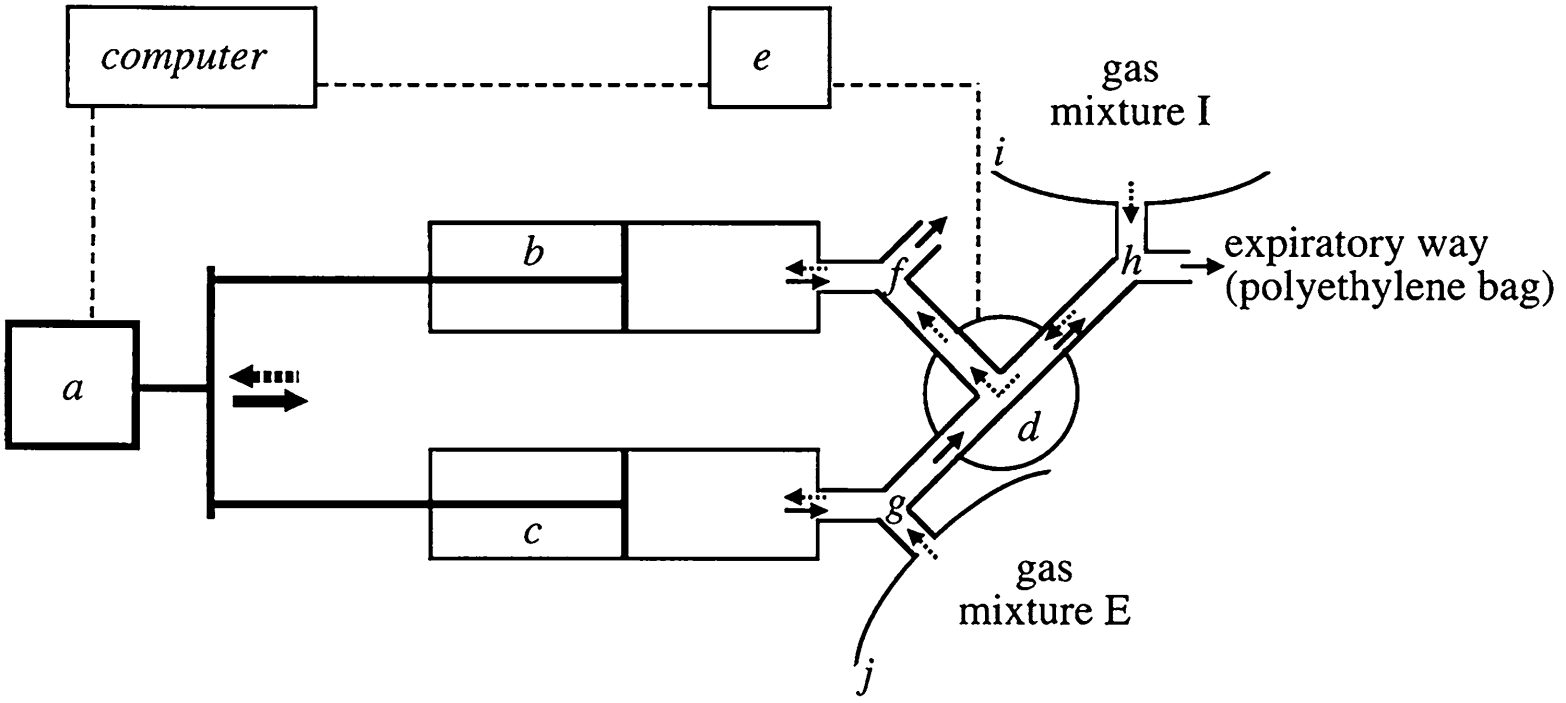
Diagram of a gas-exchange simulation system. Dotted arrows represent gas flow during inspiration and solid arrows gas flow during expiration. *a*, motion control; *b* and *c*, 3−1 syringes; *d*, pneumatic directional control valve; *e*, automated controller for directional valve; *f, g* and *h*, two-way non- rebreathing valves; *I* and *j* polyethylene bags (2000 1). Adopted from: a system to simulate gas exchange in humans to control quality of metabolic measurements (Prieur et al. 1998)

Gas infusion is the method of choice for calorimeters that use a breath-by-breath analysis, measuring inhalation and exhalation separately. In that particular case, the infusion must be combined with a realistic lung simulation for breathing **(**Fig. 9 **)** (Prieur et al. 1998), or else the calorimeter may not even function in this mode.

Unfortunately, a combined gas infusion and realistic lung simulator is not easily available, for the greater part preventing regular on-site validation of breath-by-breath calorimetry. Instead, such calorimeters may be tested only partially in their hood mode.

Gas infusion in general has the advantages of being able to achieve the broadest possible range of gas concentrations and a fast modulation of gas quantities infused. Hence, it is the method of choice for mimicking exercise.

### Parallel validation

A third validation method is realized by combining two methods, i.e., one prior validated system and the system under test. The two systems are connected to measure the identical samples for measurements, and results are compared.

In applying this parallel validation, great care must be taken to prevent interaction between systems. This must be proven, as well as showing the intact validity of the prior validated system.

Simply interconnecting two devices, one of which was at some point validated in the literature, will not suffice. A calorimeter used as reference in such a test must be validated on site, and it cannot be assumed to perform as a “gold standard” apparatus without checking its individual performance and including possible interactions.

Note that parallel validation will typically introduce the concept of biological reproducibility, using results of a participant in the parallel measurement.

### Using biological reproducibility for validation

Consider a technically validated calorimeter failing to show reproducibility for normal healthy subjects within known limits of biological variability. In that case, the technical validation method may have failed to sufficiently simulate biology, and the calorimeter is not suitable. It cannot be deemed validated. Consider a calorimeter showing reproducibility for normal healthy subjects within known limits of biological variability, though its technical validity or “level” of measured value is unknown. In this instance, the calorimeter may, or may not, be valid. It too cannot be deemed validated. Only a technically validated calorimeter also showing reproducibility for normal healthy subjects within known limits of biological variability may be considered a validated calorimeter. It must have proven to measure exact levels of EE and its ability to reproduce measurements with participants.

Repeated measurements with subjects were performed to test reproducibility (including both technical and biological variation) from the lowest level of EE, i.e., SMR (Schoffelen and Westerterp 2008), BMR (Adriaens et al. 2003), until 24-h EE (Schoffelen et al. 1997) and to the highest possible, i.e., VO_2_max (Schoffelen et al. 2017) (Table 2). The categories Sleep, OMR, BMR, 24-h EE, and Exercise showed a CV% that was smallest for high-level exercise and largest for outpatient BMR (Table 2). Although exercise had the lowest CV% of 1.2%, it has the largest standard deviation as expressed in kJ min^−1^. In contrast, SMR with its medium CV% of 2.4% showed the best absolute reproducibility, closely followed by OMR, 24-h EE and even BMR with an outpatient protocol (Table 2).

**Table 2:** Reproducibility of EE measures using repeated subject testing

Category	EE (kJ min^−1^)	SD (kJ min^−1^)	CV (%)
SMR (room)	4.6	0.11	2.4
OMR (room)	4.8	0.13	2.8
BMR (hood)	4.7	0.15	3.3
24-h EE (room)	6.8	0.13	1.9
maximal exercise, *V*O_2max_	~ 90^a^	~ 1^a^	1.2

Data are expressed as mean EE (kj min^−1^), standard deviation (kj min^−1^), and the coefficient of variation (CV%)

*EE* energy expenditure, *SMR* sleeping metabolic rate, *OMR* overnight metabolic rate, *BMR* basal metabolic rate

^a^EE values for *V*O_2_max were estimated for comparison of size only; they are not exact because during high intensity exercise, RER > 1, and hence, EE cannot be accurately determined due to the contribution of anaerobic metabolism

## Fields of application

Since the first built room calorimeter over 150 years ago, calorimeters have evolved over time from very labor intensive instruments with chemical analysis of gasses to modern automated systems with continuous gas analysis and online data processing. These technological advancements have also lead to faster response times and higher sampling resolutions, opening up the field to a wide variety of applications. Room calorimeters have been and, currently, are being, used to study human energy metabolism under all sorts of environmental conditions.

Many studies involved testing the metabolic response to different food products, with manipulation of the amount of food (Wulan et al. 2014; Rynders et al. 2017), the relative contribution of the three main substrates, i.e., carbohydrates, fats, and proteins (Chan et al. 2014; Munsters et al. 2013), or the response to the so-called thermogenic ingredients such as tea extracts (Gregersen et al. 2009) or capsaicin (red pepper extract) (Janssens et al. 2013). Since the discovery of the existence of brown adipose tissue in adult humans, thermal physiology studying human metabolism during cold (or heat) exposure has gained a new interest (Wijers et al. 2010; Chen et al. 2013; Hibi et al. 2016; Peterson et al. 2016). In addition, given our modern global society where people can be in contact 24 h a day all over the world, regularly travel through different time zones, and are exposed to screen light on their electronic devices almost constantly, the study of sleep and circadian rhythms and the effect on human metabolism have become an important field of research (Jung et al. 2011; Gonnissen et al. 2013; McHill et al. 2014). Others have studied the effect of seasons (Plasqui et al. 2003; Minghelli et al. 1991), differences between ethnicities (Dugas et al. 2009; Wulan et al. 2012), or EE during pregnancy (Gilmore et al. 2016).

The controlled environment also provides excellent opportunity for exercise studies, as modern day calorimeters have the resolution to study EE almost in real time, even in a large volume such as a room calorimeter (Robinson et al. 2015; Frost et al. 2014). Recent data have shown that even VO_2_-max testing is possible in a room calorimeter sized 18 m^3^ (Kleinloog et al. 2017). In addition, the higher time resolution better allows performing validation studies of different wearable sensors and their ability to estimate EE (Dannecker et al. 2013).

All of the above research topics are just a small fraction of published research and there are many more applications. Room calorimeters provide an ideal platform to study metabolism under strictly controlled conditions, where all food intake, temperature, humidity, lighting, and even O_2_ pressure can be regulated and the response of human EE and substrate oxidation can be measured.

## Conclusion

From the different indirect calorimetry techniques available, this review mainly focused on the open-circuit diluted flow principle, which can be applied with a facemask, ventilated hood or respiration chamber. All exhaled air is captured and diluted in a bypass flow that can be varied depending on the level of gas exchange, i.e., from sleeping metabolic rate until VO_2_max. One of the advantages is that there are no restrictions at the mouth caused by valves or flowmeters.

For bedside and exercise applications, the open-circuit diluted flow principle can be considered a modern day replacement of the full-capture calorimeters of the past. These applications have become a reality due to developments in electronics and analyser accuracy, exchanging large volumes for fast response while taking advantage of modern day whole-room calorimetric equipment. The application of indirect calorimeters is not as straightforward as the less knowledgeable user may expect. For reliable and accurate results, it requires expert know-how, suitable and frequent validation, and meticulous attention to detail. Old gold standard facts are as applicable as ever, effectively eliminating any calorimeter as being a gold standard “out of the box” and without evaluation. Effectively, there is much to be gained by studying the calorimetric principles published in the distant past.

## Abbreviations

AEE: Activity-related energy expenditure
BMR: Basal metabolic rate
DIT: Diet-induced thermogenesis
EE: Energy expenditure
SMR: Sleeping metabolic rate
